# Determinants of bed net use among older people in Nigeria: results from a nationally representative survey

**DOI:** 10.11604/pamj.2018.31.112.12627

**Published:** 2018-10-15

**Authors:** Saliu Balogun, Hakeem Yusuff, Bilkis Adeleye, Mariam Balogun, Abodunrin Aminu, Kehinde Yusuf, Prudence Tettey

**Affiliations:** 1 Menzies Institute for Medical Research, University of Tasmania, Australia; 2 Otun Centre for Health and Social Research, Lagos State, Nigeria; 3 State Ministry of Health, Abeokuta, Ogun State, Nigeria; 4 Alimosho General Hospital, Lagos, Nigeria; 5 Life without Barriers, Tasmania, Australia; 6 Centre for Research on Ageing, University of Southampton, United Kingdom; 7 College of Medical Rehabilitation Sciences, Taibah University, Madinah, Saudi Arabia

**Keywords:** Malaria, Nigeria, bed nets, older people, insecticide-treated net

## Abstract

**Introduction:**

the use of bed nets is a well-recognized and cost-effective preventive measure against malaria. However, little is known about factors associated with the use of bed nets among older people in Nigeria. Therefore, this study aimed to examine the determinants of bed net use among older Nigerian adults.

**Methods:**

data from the first wave of the Nigeria General Household Survey-Panel were used, which included 3,439 participants aged 50 years and above. Log-binomial models were used to model the association between participants' sociodemographic characteristics and the use of bed nets.

**Results:**

the frequency of bed net use was 26%. The adjusted prevalence ratio (PR) of bed net use was lower in women (PR = 0.83, 95% CI: 0.73-0.96), older age groups (60-69 years: PR=0.85, 95% CI:0.75-0.97; 70 years and above: PR = 0.80, 95% CI: 0.69-0.94), female-headed households (PR = 0.69, 95% CI: 0.53-0.89) and among those in the highest tertiles of per-capita household expenditure (PR=0.77, 95% CI: 0.66-0.90). However, the frequency of bed net use was higher among older adults residing in the rural areas (PR = 1.84, 95% CI: 1.55-2.18) and those who reported never having attended school (PR=1.15, 95% CI: 1.01-1.30).

**Conclusion:**

the prevalence of bed net use among older adults is lower compared to previously reported data for younger adults, suggesting an increased risk of the older adults of exposure to malaria. Female sex, age ≥ 60 years, level of education, economic status, and rural vs urban residence were important determinants of bed net use among older adults.

## Introduction

Malaria is a major public health problem particularly in sub-Saharan Africa (SSA) where it has significant economic, financial and health consequences [1]. For instance, in 2015 there were 214 million cases of malaria worldwide, including 438,000 which were fatal. Ninety percent of these fatal cases occurred in SSA, including more than 35% in Nigeria and the Democratic Republic of the Congo alone [2]. Malaria is endemic in Nigeria, with more than 90% of the estimated 160 million people in the country at risk. Moreover, 60% of outpatient clinic/hospital visits in Nigeria are malaria-related [3, 4]. Malaria is a parasitic disease transmitted through mosquito bites and using insecticide-treated bed nets is a well-recognized, cost-effective preventive tool against mosquitoes and thus, malaria infection. If properly used, insecticide-treated bed nets could provide almost complete protection from mosquito bites, leading to a marked reduction in all-cause mortality [5, 6]. Despite the obvious efficacy and cost-effectiveness of bed nets in preventing morbidity and mortality from malaria, the use of bed nets in several SSA countries is low [7]. Studies on malaria prevention strategies, including the use of bed nets, have focused largely on pregnant women and children under five years [8-10]. Limited data are available on the impact of malaria and prevention of malaria among older people in Nigeria, despite studies reporting that one in four of older adults reported having had recent malaria-related morbidity [11]. Recently, the World Health Organisation developed a global technical strategy aimed at reducing the incidence and mortality of malaria around the world by at least 90 percent by 2030 [2]. A major step in achieving such a target, particularly among older people in Nigeria, is to identify sub-groups of older adults with low prevention-seeking behaviour that could be integrated into malaria prevention programmes. Therefore, this study aimed to examine the determinants of bed net use among Nigerian older adults using a large nationally representative sample.

## Methods

**Data and methods:** data for men and women aged 50 years and above were analyzed from the Nigeria General Household Survey-Panel (Nigeria GHS-Panel). The Nigeria GHS-Panel is a nationally representative household survey conducted by the Nigerian National Bureau of Statistics, with support from the World Bank [12]. The survey provides a reliable estimate of key socio-economic variables for the six geopolitical zones in Nigeria [12]. The Nigeria GHS-Panel was carried out twice in each wave, once after the planting season (post-planting visit, August-October, 2010) and the other after the harvest season (post-harvest visit, February- April 2011). The Nigeria GHS-Panel survey used a multi-stage sampling technique that randomly selected 5,000 households, involving 27,533 household members. A total of 3,586 adults aged 50 and above were selected for analysis in this study. Age 50 was selected as the cut-off for old age in this study, as this threshold has been used previously in studies from other SSA countries [13-15]. The use of age 50 as the cut-point for old age is also logical given that life expectancy is low (i.e. 55 years) in SSA countries [16], with the average life expectancy at birth for people in Nigeria only being 52 years [17].

**Outcome measure: use of bed nets:** the use of bed nets was assessed based on a participant's response to the question asking whether or not they slept under a bed net in the night preceding the survey, to which participants could respond yes or no. This mode of assessing bed net use has been used in several studies in SSA countries [7, 8, 18].

**Explanatory variables:** explanatory variables considered in this study included age, place of residence (0 = urban, 1 = rural) and living arrangements (0 = co-reside with others, 1=living alone). Marital status at interview was classified into two categories (0 = married and 1 = not currently married). Education was assessed based on a participant's response to the question, “Have you ever attended school?” Response to this question was dichotomised (0 = attended school, 1 = never attended school). Female headed households (1= female and 0 = male) were defined as households in which an adult female is the sole head. Economic status was measured as tertiles of per-capita household expenditure (that is, total household expenditure divided by the household size) [19]. Household expenditure represents the total expenses paid for food and non-food items (i.e. health-related expenses, housing, electricity and other goods and services) in each household. Since the data was collected twice in each wave, the aggregated household expenditure was the average household expenditure across both visits. Household expenditure as captured in this paper is a direct measure, and is, therefore, the preferred measure for living standards, as the collection of expenditure data is more reliable than utilising a measure such as income [20].

**Data analysis:** descriptive statistics for each explanatory variable were compared for older adults who used bed nets and those who did not. We used log-binomial models to identify the socio-demographic variables that contribute to the use of bed nets [21]. At first, we assessed the unadjusted relationship between each predictor variable and use of bed net, followed by four regression models developed by stepwise addition. In the first model, sex and age groups were included as the covariates. In the second model, marital status and living arrangement were added. Education and tertiles of per-capita household expenditure were included as covariates in the third model. In the fourth and final model, place of residence and heads of household were included as potential explanatory variables. We evaluated multi-collinearity among all the predictor variables to identify possible collinearity. There was no variance inflation factor of greater than 10, suggesting no cause for multi-collinearity in the models [22]. Statistical interaction between living arrangement and socioeconomic factors (education and tertiles of per-capita household expenditure) was assessed by a test of significance of a product term. Data analysis was performed using Stata version 13 (Stata Corp LLC, College Station, Texas, USA) and P < 0.05 was considered statistically significant.

## Results

**Figure 1:**
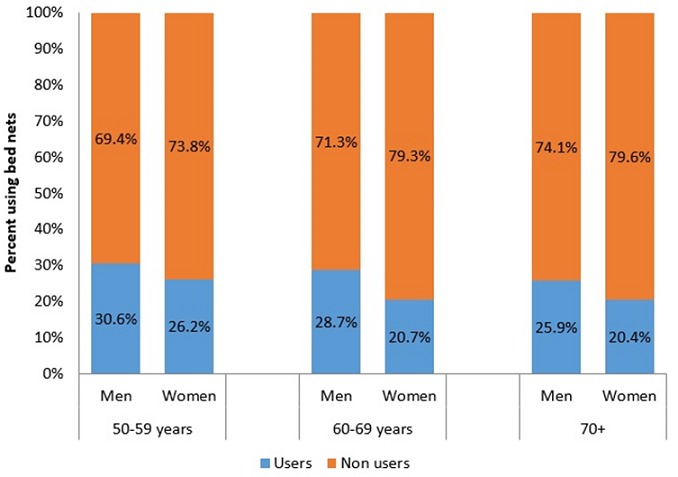
percentage of men and women using bed net stratified by age groups

**Figure 2:**
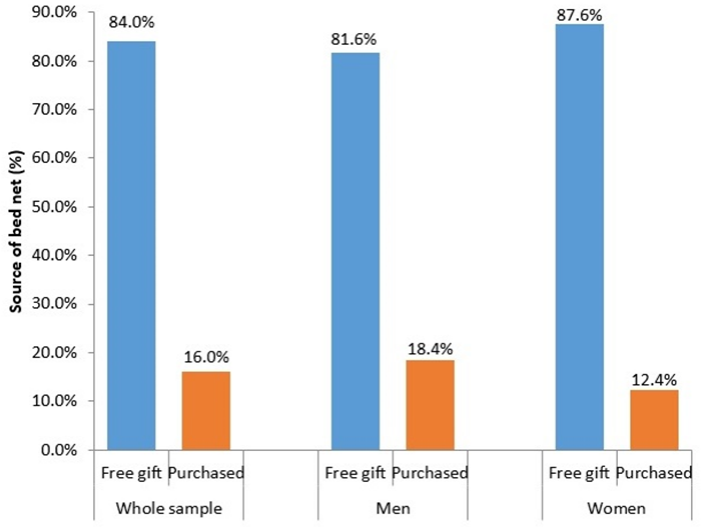
sources of bed net use among current users stratified by sex

**Descriptive characteristics of the participant and bed net use:** Table 1 presents descriptive characteristics of the participants stratified by use of bed nets. Participants who used a bed net were younger (60.8±10.1 vs. 62.0±10.2 in non-users, P = 0.001) and the use of bed nets decreased with increasing age groups (50-59 years: 28.5%, 60-69 years: 25.1%, 70 years and above: 23.7%, P = 0.002). The use of bed nets was lower among women (23.3% vs. 28.8% in men, P < 0.001), among participants who were not currently married (21.8% vs. 27.7% in currently married, P = 0.001), among older adults living alone (15% vs. 27% among those co-residing with others, P < 0.001), and among those who reported having attended school (24% vs. 28.4% in never attended school, P = 0.003). Bed net use was significantly (P < 0.001) higher among older adults in the lowest tertiles (33%) of per-capita household expenditure compared to those in the middle (27%) and highest tertiles (19%). Furthermore, the use of bed nets was significantly lower among older adults residing in urban areas (15.3% vs. 30.9% in rural areas, P < 0.001) and in female-headed households (16.5% vs. 28% in male-headed households, P < 0.001). Figure 1 shows the percentage of men and women using bed nets stratified by age group. For both men and women, the use of bed nets decreased with increasing age (P < 0.05). However, among men and women of the same age group, the use of bed net was lower in women compared to men. The source of bed nets stratified by sex is presented in Figure 2. Overall, 84% of the participants obtained their bed net as a free gift compared to 16% who purchased their bed nets. The proportion of participants who purchased their bed net was higher in men (18.4%) compared to women (12.4%).

**Table 1:** descriptive statistics of explanatory variables stratified by use of bed net

Variables	Total	Non-users		Users		*P*-value
		n	% or mean (SD)	N	% or mean (SD)	
**Mean age (SD)**	3,439	2,535	62.0 (10.2)	904	60.8 (10.1)	0.001
**Age groups**						
50-59 years	1,554	1,111	71.5	443	28.5	
60-69 years	1,070	802	75.0	268	25.1	0.022
70+ years	815	622	76.3	193	23.7	
**Sex**						
Men	1,871	1,332	71.2	539	28.8	<0.001
Women	1,568	1,203	76.7	365	23.3	
**Marital status**						
Married	2,624	1,896	72.3	728	27.7	0.001
Not currently married†	809	633	78.2	176	21.8	
**Living arrangement**						
Co-reside with others	3,246	2,371	73.0	875	27.0	<0.001
Living alone	193	164	85.0	29	15.0	
**Ever attended school**						
Attended school	1,689	1,284	76.0	405	24.0	0.003
Never attended school	1,732	1,240	71.6	492	28.4	
**Socioeconomic status‡**						
Lowest	1,098	736	67.0	362	33.0	
Second	1,098	800	72.9	298	27.1	<0.001
Highest	1,097	886	80.8	211	19.2	
**Place of residence**						
Urban	1,008	854	84.7	154	15.3	<0.001
Rural	2,431	1,681	69.2	750	30.9	
**Head of household**						
Male-headed household	2,930	2,110	72.0	820	28.0	<0.001
Female-headed household	509	425	83.5	84	16.5	

† Widowed/widower, divorced, separated or never married;

‡ Tertiles of per-capita household expenditure

**Determinants of bed net use:** Table 2 shows the association between use of bed net and each explanatory variable. The gross effects model (Table 2, column 2) shows the independent association of each explanatory variable with use of bed nets. In model 1, the prevalence of bed net use was lower in women (PR = 0.80, 95% CI: 0.71-0.90) compared to men. Furthermore, the prevalence of bed net use was lower among participants who were in the 60-69 years age group (PR = 0.87, 95% CI: 0.77-0.99) and those in the 70 years and above category (PR = 0.82, 95% CI: 0.71-0.95) compared to those who were in the 50-59 years age group. This pattern remained after adjusting for marital status and living arrangement (model 2, Table 2). The prevalence of bed net use among older adults living alone (PR = 0.80, 95% CI 0.55-1.16) became non-significant after adjusting for education and tertiles of per-capita household expenditure (model 3, Table 2). In the fully adjusted model, the frequency bed net use was lower in women (PR = 0.83, 95% CI: 0.73-0.96), older age groups (60-69 years: PR = 0.85, 95% CI:0.75-0.97; 70 years and above: PR = 0.80, 95% CI: 0.69-0.94), female-headed households (PR = 0.69, 95% CI: 0.53-0.89) and among older adults in the highest tertiles of per-capita household expenditure (PR = 0.77, 95% CI: 0.66-0.90). However, the prevalence of bed net use was higher among older adults residing in the rural areas (PR = 1.84, 95% CI: 1.55-2.18) and those who were not educated (PR = 1.15, 95% CI: 1.01-1.30). The finding that the association between bed net use and living arrangement became non-significant after adjusting for education and tertiles of per-capita household expenditure suggests that these economic indices may play a role in the association between living arrangement and the use of bed nets. Thus, we assessed the multiplicative interaction between living arrangement and education, to examine whether education modifies the association between living arrangement and bed net use. We also performed a similar test for interaction between living arrangement and tertiles of per-capita household expenditure. We found no evidence for an interaction between living arrangement and household economic status (P-value _interaction_ = 0.76). However, there was a significant interaction between living arrangement and education (P-value _interaction_ = 0.001), such that the use of bed nets was significantly lower among older adults living alone and who were not educated (PR = 0.40, 95% CI: 0.21-0.77, P = 0.006), whereas no association was found among older adults who were educated and living alone (PR = 1.50, 95% CI: 0.96-2.36, P = 0.077).

**Table 2:** prevalent ratios (PR) of bed net use among older adults aged 50 years and above in Nigeria

Variables	Univariable model PR (95% CI)	Model 1 PR (95% CI)	Model 2 PR (95% CI)	Model 3 PR (95% CI)	Model 4 PR (95% CI)
**Sex**					
Male	1.00	1.00	1.00	1.00	1.00
Female	0.81 (0.72-0.91)	0.80 (0.71-0.90)	0.83 (0.73-0.94)	0.79 (0.69-0.90)	0.83 (0.73-0.96)
**Age groups**					
50-59years	1.00	1.00	1.00	1.00	1.00
60-69 years	0.88 (0.77-1.00)	0.87 (0.77-0.99)	0.89 (0.78-1.01)	0.85 (0.75-0.97)	0.85 (0.75-0.97)
70 years and above	0.83 (0.72-0.96)	0.82 (0.71-0.95)	0.85 (0.73-0.98)	0.80 (0.64-0.94)	0.80 (0.69-0.94)
**Marital status**					
Currently married	1.00		1.00	1.00	1.00
Not currently married†	0.78 (0.68-0.91)		0.95 (0.80-1.12)	0.97 (0.81-1.15)	1.13 (0.94-1.36)
**Living arrangement**					
Co-reside with others	1.00		1.00	1.00	1.00
Living alone	0.61 (0.43-0.87)		0.69(0.48-1.00)	0.80 (0.55-1.16)	0.87 (0.59-1.26)
**Education**					
Attended school	1.00			1.00	1.00
Never attended school	1.18 (1.06-1.32)			1.22 (1.08-1.39)	1.15 (1.01-1.30)
**Socioeconomic status‡**					
Lowest	1.00			1.00	1.00
Second	0.82 (0.73-0.94)			0.85 (0.75-0.97)	0.92 (0.81-1.05)
Highest	0.58 (0.50-0.68)			0.64 (0.55-0.75)	0.77 (0.66-0.90)
**Residence**					
Urban	1.00				1.00
Rural	2.02 (1.73-2.36)				1.84 (1.55-2.18)
**Head of household**					
Male headed household	1.00				1.00
Female headed household	0.59 (0.48-0.72)				0.69 (0.53-0.89)
*Model sample size*		34,39	3,433	3,274	3,274

*Data in bold indicates statistical significance at *P* < 0.05

Model 1: Adjusted for age and sex

Model 2: Model 1 + marital status and living arrangement

Model 3: Model 2 + education and socioeconomic status

Model 4: Model 3 + place of residence and head of households

† Widowed/widower, divorced, separated or never married

‡ Tertiles of per-capita household expenditure

## Discussion

This is the first study, to our knowledge, investigating the socio-demographic determinants of bed net use among older people in Nigeria. We found that the prevalence of bed net use was significantly lower among women compared to men, irrespective of age group. Furthermore, the use of bed nets was lower in female-headed households, among older participants, and among participants in the middle and highest tertiles of per-capita household expenditure. However, older adults residing in rural areas and those who were not educated had a significantly higher prevalence of bed net use. Our finding that 26% of the older adults slept under a bed net on the night prior to the survey was lower than the 44% previously reported for women of reproductive age in Nigeria [8], suggesting an increased susceptibility to malaria via exposure to a mosquito bite in this sample. Among participants who slept under a bed net, 84% of the bed nets were obtained as a free gift, reflecting the positive impact of the free bed net distribution programs by the government and other non-governmental organisations as part of a massive campaign to reduce malaria-related morbidity [23]. Of concern is the finding that the prevalence of bed net use decreased with age, with the prevalence of bed net use lowest in the oldest age group category (70 years and above). Malaria is associated with fever, loss of appetite, headache and other flu-like symptoms that could compromise the health and quality of life of the older adults. In Nigeria, where there is no universal health and welfare system, this lower frequency of bed net use in the oldest age group category could be exposing this group of older people to malaria. Our finding that the prevalence of bed net use was significantly lower among women is inconsistent with several studies among young adults which showed that the use of bed nets was significantly higher among women compared to men [7, 24]. Potentially, the reason for the lower prevalence of bed net use among older women could be related to the transition in their life cycle. For instance, sleeping alongside young children is common for mothers in most SSA countries. Because children, particularly infants, are more likely to sleep under bed nets, it is possible that a greater proportion of younger women sleep under bed nets because of their child [18, 25]. However, as children grow older, the use of bed net may be lower among older women. In the 2006 Malaria Indicator survey from Ethiopia, women constitute a higher proportion of participants below aged 50 who slept under a bed net in the previous night compared to men [24]. However, from aged 50 and above, more men compared to women slept under the bed net. This finding reflects in our results and it suggests an increased risk of older women to malaria compared to men.

Education modified the association between living arrangement and use of bed net, such that the prevalence of bed net use was lower among older adults who were living alone and had no education, whereas living arrangement had no impact on bed net use among older adults who were educated. Since living alone in old age is associated with poverty and poor health risk [26], it is possible that bed net use was significantly lower among older people who were not educated and living alone because the lack of education conferred additional risk besides the consequences of living alone. There was no evidence of an association between marital status and the use of bed nets in the fully adjusted model, suggesting that the former may be confounded by other factors. The finding that the prevalence of bed net use was higher among older people in the lower tertiles of per-capita household expenditure is unexpected. This finding is inconsistent with results from Uganda and Tanzania which showed that the use of bed nets was higher among individuals in wealthy households compared to those from poor households [27]. However, our result is consistent with prior studies among women and children in Nigeria, which showed that, although ownership of bed net was higher in wealthy households, the use of bed net was significantly higher among individuals in poorer households compared to those from wealthy households [8]. The reasons for this observation is not clear but may be related to changes in health behaviour as a result of self-perceived vulnerability to malaria [8]. Healthcare utilization in Nigeria is mostly by out-of-pocket payment [28] and individuals in poor socio-economic status may be less likely to access healthcare because of their low financial capacity. Hence, it is possible that individuals in the lower tertiles of per-capita household expenditure adopted a preventive health measure against malaria by using a bed net because of their self-perceived vulnerability. Furthermore, our finding that the prevalence of bed net use was higher among older adults who were not educated has been previously reported in Nigeria and Uganda and was related to perceived vulnerability to malaria [8, 29, 30]. Taken together, the findings that the prevalence of bed net use was significantly higher among older adults in the lower tertiles of per-capita household expenditure among those with no education are encouraging as it demonstrates positive health behaviour against malaria among older adults that could be considered a high-risk group. Interestingly, we found that the use of bed nets was lower among individuals living in the female-headed households compared to those living in a male-headed household. This observation is consistent with a prior study from Kenya and the authors attributed their findings to better education and economic status among male-headed households [31]. However, adjusting for education and socioeconomic status does not explain the lower prevalence ratio of bed net use in female-headed households in our sample. Besides, we found no evidence for an interaction between head of household and education (or socioeconomic status) in predicting use of bed nets (data not shown), suggesting that additional variables beyond those considered in this study are needed to fully explain the lower prevalence of bed net use in female-headed households. Our finding that the use of bed nets was significantly higher among older people residing in the rural areas is encouraging, given that malaria parasitemia has been shown to be higher in rural areas [32].

This study has a number of strengths which include: large sample size; a very high response rate and, the use of a nationally representative dataset, which increases the generalizability of our findings. However, this study has a number of limitations. First, there was no data collected about the ownership, as distinct from recent use, of bed nets. This variable may be particularly important to investigate, as access to bed nets may facilitate their use. Hence, we are unable to determine whether the use of bed nets was merely a function of ownership or if there are sizeable proportions of the populations who own but do not use bed nets. Second, the use of bed nets was self-reported and may be subjected to reporting biases. Third, data about other malaria preventive measures such as mosquito coils, canned insect spray, and/or indoor residual spraying with insecticide were not collected in the Nigeria GHS-Panel, and so we were unable to determine whether some of the participants were using some of this other malaria preventive measures rather than bed nets. Fourth, data used in this study was collected between February and April; it is possible that the prevalence of bed net use could be higher if the data were collected at a different time point. However, a prior study among pregnant women and children in 15 African countries found no evidence of an association between season and the use of bed nets [18]. Lastly, due to the cross-sectional nature of this study, we cannot establish causal relationships between use of bed net and the various exposure variables. Despite these limitations, this study provides the first nationally representative data on the determinants of bed net use among older people in Nigeria. Findings from this study could be relevant in the design of malaria prevention strategies among Nigerian older adults as it suggests sub-group of older adults with a lower prevalence of bed net use that could be targeted for malaria prevention.

## Conclusion

The prevalence of bed net use among older adults is lower compared to previously reported data for younger adults, suggesting an increased susceptibility of older adults to malaria exposure. Sex, increasing age, education, economic status and place of residence were important determinants of bed net use.

### What is known about this topic

- Malaria is endemic in Nigeria, affecting individuals of all age groups;
- Studies on malaria prevention strategies have largely focused on pregnant women and children under five years;
- Little is known about malaria preventive behaviours among older people in Nigeria.

### What this study adds

- The use of bed nets among older adults is lower compared to previously reported data for younger adults;
- Sex, increasing age, education, economic status and place of residence were important determinants of bed net use among older people.

## Competing interests

The authors declare no competing interest.
